# Direct Hospitalisation Costs of End-of-Life Cancer Care: Comparison Between Usual Care and Palliative Care Consultation

**DOI:** 10.3390/healthcare14142210

**Published:** 2026-07-21

**Authors:** Pattamas Ruanpech, Sanhapan Wattanapisit, Apichai Wattanapisit

**Affiliations:** 1 Palliative Care Unit, Thasala Hospital, Thasala, Nakhon Si Thammarat 80160, Thailand; 2 Department of Social Medicine, Thasala Hospital, Thasala, Nakhon Si Thammarat 80160, Thailand; 3 Department of Family and Preventive Medicine, Faculty of Medicine, Prince of Songkla University, Hat Yai, Songkhla 90110, Thailand

**Keywords:** cancer, consultation, cost, end-of-life care, hospitalisation

## Abstract

**Purpose:** This study aimed to compare direct hospitalisation costs for patients with cancer during terminal hospitalisation between those receiving palliative care consultation and those receiving usual care. **Methods:** A retrospective medical record review was conducted to obtain clinical information and cost data for patients with cancer during terminal hospitalisation. Hospitalisation costs between the palliative care and usual care groups were compared using analytical statistics. Effect sizes for cost comparisons were presented as median differences. **Results:** A total of 154 and 112 patients were classified into the palliative care group and the usual care group, respectively. Patients in the palliative care group had a longer length of hospital stay than those in the usual care group (8 days vs. 3 days, *p* < 0.001). Compared with usual care, patients receiving palliative care had lower utilisation of the intensive care unit (ICU) (8.4% vs. 44.6%, *p* < 0.001), invasive mechanical ventilation (4.5% vs. 36.6%, *p* < 0.001), cardiopulmonary resuscitation (1.9% vs. 8.9%, *p* = 0.020), and vasopressors and inotropes (7.1% vs. 25.9%, *p* < 0.001). Total hospitalisation costs did not differ significantly between palliative care and usual care groups (27,910.8 vs. 24,216.4 Thai Baht (THB), *p* = 0.305). However, the palliative care group had significantly lower daily hospitalisation costs (3587.4 vs. 6918.2 THB/day, *p* < 0.001). Median daily costs were lower in the palliative care group for breast cancer (3326.8 vs. 8049.0 THB/day; median difference −4390.1, *p* = 0.016), colorectal cancer (3422.8 vs. 5595.0 THB/day; median difference −2500.7, *p* = 0.008), hepato-pancreatico-biliary cancer (3611.7 vs. 5097.0 THB/day; median difference −1740.0, *p* = 0.004), and lung cancer (3435.4 vs. 7998.7 THB/day; median difference −3896.5, *p* < 0.001). **Conclusions:** Palliative care consultation was associated with lower direct daily hospitalisation costs during end-of-life cancer care.

## 1. Introduction

Cancer is a major burden worldwide and the leading cause of death in Thailand. Cancer-related deaths have steadily increased in recent years [1,2]. Patients in the late stages of cancer often experience significant physical and psychological distress, frequently leading to terminal hospitalisation. Several studies have shown that hospitals are a major setting for end-of-life care across various contexts [3,4,5,6,7,8]. Hospitalisation during the end-of-life stage frequently involves intensive medical interventions, medications, and resource utilisation, leading to escalating healthcare expenditures [9,10]. Globally, the economic burden is substantial. One study estimated that the cost of 29 cancer types across 204 countries would result in a cumulative cost of USD25.2 trillion between 2020 and 2050, representing approximately 0.55% of the global gross domestic product [11].

A widely recognised approach to managing terminal cancer is palliative care. Palliative care aims to alleviate suffering and enhance the overall quality of life for both patients and their families [12]. Integrating palliative care into the management of terminal illnesses helps to lower healthcare costs by reducing reliance on unnecessary treatments [13]. Studies from many countries suggest that such interventions provide significant economic benefits, with inpatient palliative care programmes effectively decreasing the overall cost of hospital stays for patients at the end of life [14,15,16,17,18]. These findings are consistent with previous studies in Thailand, which demonstrate that hospital-based palliative care significantly reduces healthcare costs, particularly during the end-of-life period among patients with cancer [19,20,21,22,23]. Specifically, a study conducted at a Thai university hospital reported that palliative care for patients with end-stage liver, bile duct, and lung cancers resulted in cost savings of 16,669 Thai Baht (THB) per patient [20]. Furthermore, palliative care was associated with a significant reduction in invasive treatments, such as intubation, surgery, and chemotherapy, as well as laboratory testing and intensive care unit (ICU) admissions [19,20,22,23].

In Thailand, the national ‘Service Plan’ policy has integrated palliative care for patients with life-threatening illnesses, including terminal-stage cancers, across all levels of hospitals since 2017 [24]. However, the availability of palliative care specialists remains limited and is primarily concentrated in tertiary hospitals. Consequently, palliative care in district hospitals is commonly delivered by trained family physicians who lead palliative care teams. Compared to tertiary hospitals, district hospitals also face distinct challenges, including lower staffing levels, limited palliative care infrastructure, and restricted access to treatment resources. To date, there is a lack of evidence regarding the cost analysis of hospital-based palliative care for end-of-life cancer care in district hospital settings.

The main objective of this study was to compare direct costs for patients with cancer during terminal hospitalisation between those receiving hospital-based palliative care and those receiving usual care without palliative care consultation. Additionally, this study aimed to characterise patterns of terminal cancer care within the hospital and to analyse differences in medical expenses before and after palliative care consultation.

## 2. Methods

### 2.1. Study Design and Ethical Approval

A retrospective medical record review was conducted. The researchers retrospectively analysed data from paper-based and electronic medical records of patients with cancer during terminal hospitalisation between 1 January 2021 and 31 December 2024. This study followed Strengthening the Reporting of Observational Studies in Epidemiology (STROBE) statement and Consolidated Health Economic Evaluation Reporting Standards 2022 (CHEERS 2022) [25,26]. The study protocol was approved in accordance with the Declaration of Helsinki by the Human Research Ethics Committee of the Nakhon Si Thammarat Provincial Public Health Office (Approval No. 205/2568). Informed consent was not required for this retrospective study of deceased patients, as approved by the Human Research Ethics Committee, and all data were anonymised prior to analysis.

### 2.2. Setting and Participants

This study was carried out at Thasala Hospital, a 300-bed district hospital in southern Thailand. The hospital serves a population of approximately 180,000 in the district and surrounding areas. It provides oncological services ranging from surgical interventions to chemotherapy for selected malignancies, supported by an ICU for complex cases. The hospital’s palliative care system, established in 2017, is managed by a palliative care team consisting of family physicians, nurses, and a pharmacist. The team focuses on patients with life-threatening illnesses, particularly those with advanced-stage cancer. Its primary objective is to alleviate symptoms and enhance quality of life. This is achieved through a consultation-based system that integrates palliative care with usual care across outpatient, inpatient, and home care services.

All patients with cancer who died during terminal hospitalisation between 1 January 2021 and 31 December 2024 were eligible for the study. The study included Thai nationals aged 18 years or older who were identified through the death registry with a primary cause of death attributed to malignant neoplasms, defined by the 10th revision of the International Classification of Diseases (ICD-10) codes C00–C97, and whose hospital records confirmed a discharge status of ‘dead’, with malignancy listed as either the principal diagnosis or a comorbidity. Patients were excluded if their medical records contained insufficient or missing data regarding key parameters required for the study.

### 2.3. Data Collection

Data were collected through a retrospective chart review in accordance with the eligibility criteria. Potential cases were identified by retrieving hospital numbers from the death registry within the electronic health record system, filtered using predefined ICD-10 codes and study-specific inclusion and exclusion criteria. Relevant data were extracted from both paper-based records (before the implementation of inpatient electronic medical records) and electronic medical records. The extracted data were systematically recorded in an Excel-based data collection form developed for this study.

The variables collected encompassed a broad range of patient information, including demographic and clinical characteristics such as sex, age, religion, healthcare insurance, cancer type, and length of hospital stay. Clinical interventions and resource utilisation were also recorded, including ICU admission and duration, use and duration of invasive mechanical ventilation, cardiopulmonary resuscitation (CPR) events, surgical procedures, chemotherapy, and administration of inotropic agents.

Palliative care consultation was operationally defined as a documented request in the medical record by the attending physician for the palliative care team consultation, or a recorded consultation with a trained family physician for palliative care co-management. This definition focused exclusively on consultations during the current hospitalisation, regardless of whether the patient had received palliative care prior to admission. Patients who received treatment from the attending physician without a documented, formal consultation with the palliative care team were classified into the usual care group. The post-palliative consultation period was defined as the interval from the date the palliative care consultation was recorded until the date of death. Palliative care-related variables were captured, specifically the receipt of palliative care consultation and the duration of palliative care involvement. Comprehensive healthcare cost data were also collected, including total hospitalisation expenses, healthcare costs incurred before and after palliative care consultation, and categorised service costs. These categories comprised laboratory services, radiological services, medical equipment, pharmaceutical and medical supplies, nursing services, room and food fees, medical fees, blood bank services, ICU services, surgical and anaesthesia services, and rehabilitation services. The daily cost for each admission was calculated by dividing the total cost by the total length of stay indicated in the medical records. Similarly, category-specific daily costs were determined by dividing the total cost within that specific category by the total length of stay. Hospitalisation costs incurred from the date of the recorded palliative care consultation until the patient’s death were defined as post-palliative care consultation costs. From a hospital perspective, direct medical costs were collected from hospital billing records using a bottom-up approach. All costs were presented in nominal THB corresponding to the year of the relevant admission (2021–2024) and were not adjusted for inflation.

### 2.4. Data Analysis

Data were analysed using the statistical software R, version 4.5.0 (RStudio, Boston, MA, USA). Descriptive statistics were used to summarise the demographic and clinical characteristics of the palliative care and usual care groups. Comparisons between the palliative care and usual care groups were performed.

Categorical variables, including sex, religion, healthcare insurance, cancer type, ICU utilisation, use of invasive mechanical ventilation, CPR events, surgical treatments, and use of inotropic agents, are presented as frequencies and percentages. Continuous variables, including age, length of hospital stay, duration of ICU admission, duration of invasive mechanical ventilation, and healthcare costs, were tested for normality using the Kolmogorov–Smirnov test and are reported as means with standard deviations (SD) or medians with interquartile ranges (IQR), depending on data distribution.

For inferential analyses, the chi-square test or Fisher’s exact test was used to examine differences in categorical variables between the two groups. For continuous variables, the independent *t*-test or Mann–Whitney U test was applied to compare costs between patients receiving palliative care and those receiving usual care. Additionally, the Mann–Whitney U test was employed to analyse changes in costs before and after palliative care consultation among patients who received their palliative care consultation during the terminal hospitalisation.

Mean or median differences were calculated to present effect sizes for cost comparisons. The daily cost distribution groups were presented as percentages with 95% confidence intervals. Within the palliative care group, total daily hospitalisation costs before and after palliative care consultation were compared. Statistical significance was defined as *p* < 0.05.

## 3. Results

**Figure 1:**
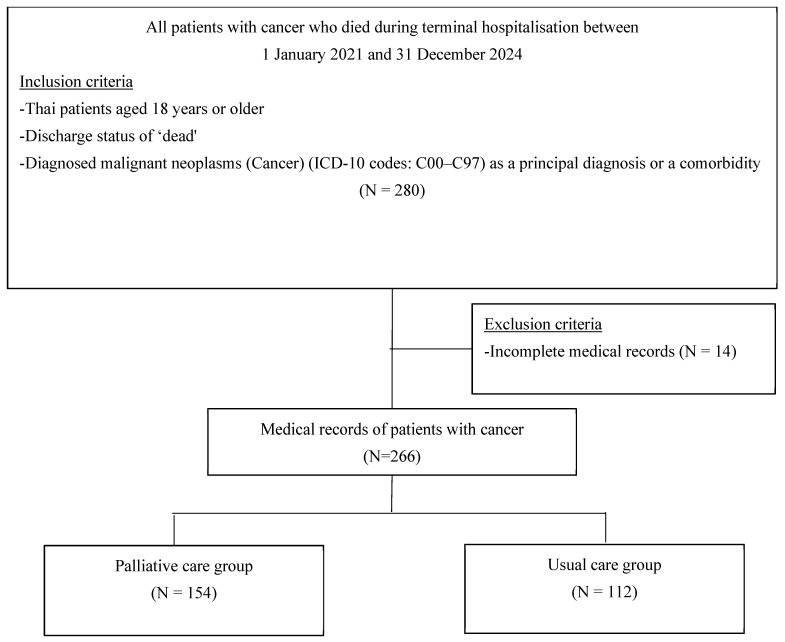
Flow diagram.

A total of 280 records of patients with cancer who died during terminal hospitalisation between 1 January 2021 and 31 December 2024 were identified. Fourteen records were excluded due to incomplete medical data, leaving 266 records included in the analysis. Of these, 154 patients were categorised into the palliative care group, and 112 patients into the usual care group (Figure 1).

Most patients were male (66.9%), with a mean age of 65.1 ± 12.8 years. The majority were Buddhist (86.5%). The Universal Coverage Scheme was the main type of health insurance (65.4%). Lung cancer was the most common cancer diagnosis (30.8%). Table 1 summarises the demographic characteristics and cancer diagnoses of patients in the palliative care and usual care groups. There were no statistically significant differences in baseline characteristics between the two groups.

Patients in the palliative care group had a longer length of hospital stay compared with those in the usual care group (8 days vs. 3 days, *p* < 0.001). Compared with the usual care group, patients receiving palliative care had lower utilisation of the ICU (8.4% vs. 44.6%, *p* < 0.001), invasive mechanical ventilation (4.5% vs. 36.6%, *p* < 0.001), CPR (1.9% vs. 8.9%, *p* = 0.020), and vasopressors and inotropes (7.1% vs. 25.9%, *p* < 0.001). In addition, the palliative care group had a significantly lower daily hospitalisation cost (3587.4 THB vs. 6918.2 THB, *p* < 0.001). The palliative care group incurred higher total hospitalisation costs than the usual care group but the difference was not statistically significant (27,910.8 vs. 24,216.4 THB, *p* = 0.305) (Table 2).

When stratified by cancer type, median daily costs were lower in the palliative care group. Statistically significant differences were observed for breast cancer (3326.8 THB/day vs. 8049.0 THB/day; median difference −4390.1 THB/day, *p* = 0.016), colorectal cancer (3422.8 THB/day vs. 5595.0 THB/day; median difference −2500.7 THB/day, *p* = 0.008), hepato-pancreatico-biliary cancer (3611.7 THB/day vs. 5097.0 THB/day; median difference −1740.0 THB/day, *p* = 0.004), and lung cancer (3435.4 THB/day vs. 7998.7 THB/day; median difference −3896.5 THB/day, *p* < 0.001) (Table 3).

Compared with similar patterns of service utilisation, the median daily cost of room and food was higher in the palliative care group (1066.4 THB/day vs. 400.0 THB/day; median difference 371.9 THB/day, *p* < 0.001). However, daily costs for other services were lower in the palliative care group. Significantly lower daily costs were observed for laboratory services (−417.4 THB/day, *p* < 0.001), radiological services (−186.7 THB/day, *p* < 0.001), medical equipment (−663.5 THB/day, *p* < 0.001), pharmaceuticals (−327.4 THB/day, *p* < 0.001), nursing services (−346.7 THB/day, *p* < 0.001), blood transfusion services (−310.6 THB/day, *p* < 0.001), and ICU services (−518.5 THB/day, *p* < 0.001) (Table 4).

**Figure 2:**
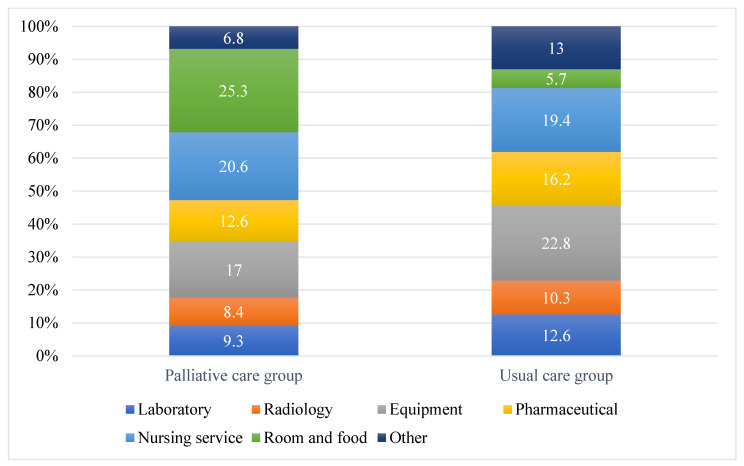
Percentage distribution of daily hospitalisation costs by service category in the palliative care and usual care groups.

The largest proportion of daily costs in the palliative care group was attributable to room and food (25.3%), followed by nursing services (20.6%). In the usual care group, the largest cost component was medical equipment (22.8%), followed by nursing services (19.4%) (Figure 2).

Within the palliative care group, differences in daily costs before and after palliative care consultation are presented in Table 5. Following palliative care consultation, daily costs for medical equipment (202.5 THB/day, *p* < 0.001) and nursing services (135.8 THB/day, *p* < 0.001) were higher compared with the period before consultation. In contrast, total daily hospitalisation costs (−528.7 THB/day, *p* = 0.008), laboratory costs (−281.6 THB/day, *p* < 0.001), and radiological costs (−211.6 THB/day, *p* < 0.001) were significantly lower after palliative care consultation.

## 4. Discussion

This study compared direct costs of terminal hospitalisation among patients who died from cancer between those who received palliative care consultation and those who did not. Overall, patients in the palliative care group had longer hospital admissions than those in the usual care group. However, total hospitalisation costs did not differ significantly between patients who received palliative care consultation and those who did not. Notably, daily hospitalisation costs were significantly lower in the palliative care group. When analysed by cancer type, daily costs among patients with breast, colorectal, hepato-pancreatico-biliary, and lung cancers were significantly lower in the palliative care group. Most service-related costs, including laboratory, radiology, medical equipment, pharmaceuticals, nursing services, blood transfusion, and ICU services, were significantly lower in the palliative care group, with the exception of room and food costs, which were higher. Furthermore, palliative care consultation was associated with a significant reduction in the utilisation of ICU, invasive mechanical ventilation, CPR, and vasopressors and inotropes. Within the palliative care group, palliative care consultation was associated with reductions in total daily costs as well as laboratory and radiological costs. In contrast, daily costs related to medical equipment and nursing services increased slightly following palliative care consultation. Direct medical costs in this study were analysed using actual values without inflation adjustment because both groups were compared head-to-head within identical timeframes, exposing them to the same economic conditions. Furthermore, Thailand’s Medical Consumer Price Index remained highly stable during 2021–2024 (ranging from 97.35 to 100.11), rendering any inflation adjustment negligible [27].

In this study, slightly more than half of the patients received palliative care consultation, reflecting both progress and persistent barriers to palliative care delivery in district hospital settings. Barriers commonly reported in such settings include limited national policy implementation, inadequate access to essential palliative medicines, and workforce shortages [28]. Although palliative care expansion remains constrained across many low- and middle-income countries (LMICs), Thailand has achieved an advanced level of palliative care development in recent global rankings [29]. Palliative care has been integrated into national health policy, with universal coverage and dedicated palliative care services established in hospitals nationwide [24]. Nevertheless, the quality and consistency of service delivery remain areas requiring ongoing evaluation and systematic improvement. Shortages of palliative care specialists, limited access to essential equipment such as syringe drivers, and inconsistent availability of analgesics persist, particularly in district hospitals compared with urban tertiary centres [24,30]. Beyond systemic barriers, distinct clinical factors and decision-making dynamics likely influenced both consultation rates and cost differences. Patients not referred to palliative care may have experienced more acute deterioration, uncertain prognoses, or intensive treatment goals that drove up expenditures. Conversely, those referred likely benefited from clearer recognition of dying, documented goals-of-care discussions, and decisions to avoid burdensome interventions. These differing clinical trajectories and care goals, rather than service availability alone, may partly explain the lower resource utilisation in the palliative care group.

Unlike high-income countries, where end-of-life care is commonly delivered in dedicated hospice facilities, Thailand has adapted hospice-like services within hospital-based settings by setting aside nicer rooms for terminal inpatients, offering outpatient consultations, or providing regular home care. These services have adapted to community-based care and some hospice-like institutions [30]. The present study was conducted in a large district hospital with oncological services, an intensive care unit, and an established palliative care team, offering relatively better resources than many district hospitals. Despite this, infrastructural limitations remained, with therapeutic capacity restricted to selected cancer types and procedures. In addition, the palliative care team relied primarily on trained family physicians and part-time palliative nurses, in the limited specialist palliative physicians. Consequently, both oncological and palliative services were less comprehensive than those available in tertiary hospitals. Notably, despite these limitations, 57.9% of patients who died from cancer during hospitalisation in this study received palliative care. This proportion is higher than that reported in previous studies conducted in regional and university hospitals in Thailand, where most patients with cancer did not receive palliative care [20,21,22]. This contrast highlights continued underutilisation of palliative care in larger institutional settings and suggests that district hospitals may play a critical role in improving access to end-of-life care.

Direct hospitalisation costs during terminal admission are an important outcome because they reflect the intensity of resource use at the end of life and have direct implications for hospital and health system budgets. Although total hospitalisation costs did not differ significantly between the palliative care and usual care groups, costs were slightly higher in the palliative care group, largely attributable to longer lengths of hospital stay. This finding may pose challenges when communicating palliative care value at the policymaking and financing levels. Total hospitalisation costs and daily hospitalisation costs capture different aspects of care. Total hospitalisation costs reflect the cumulative resource use over an entire admission and are strongly influenced by length of stay, whereas daily hospitalisation costs reflect the intensity of care delivered per unit time and are less confounded by differences in length of stay between groups. Previous studies have reported inconsistent associations between palliative care and length of hospitalisation [31,32,33]. In addition, a 2020 Cochrane review found inconclusive evidence regarding the cost-effectiveness of hospital-based palliative care, while demonstrating modest improvements in health-related quality of life, symptom control, and patient satisfaction [34]. From this perspective, palliative care adds value beyond direct cost reduction. In the present study, lower utilisation of ICUs and mechanical ventilators among patients receiving palliative care further suggests indirect resource benefits, allowing critical care resources to be reallocated to patients with potentially curative needs. Moreover, this reduced use of invasive interventions may reflect more appropriate, goal-concordant care.

When daily hospitalisation costs were examined, palliative care was associated with significant cost reductions. These findings are consistent with studies from Canada, the Czech Republic, and Thailand, which demonstrated that palliative care significantly reduced daily hospital costs for patients with cancer and other terminal illnesses. Higher levels of palliative care involvement and earlier consultation have been associated with greater cost reductions [16,17,22]. Patients receiving palliative care in this study was associated with fewer invasive interventions compared with those receiving usual care. These findings align with evidence from studies conducted in North America and Thailand, demonstrating that palliative care integration reduces aggressive treatments and ICU utilisation at the end of life [15,16,17,19,20,22].

Palliative care consultation was associated with reductions in more than 50% in direct daily hospitalisation costs for certain cancers, particularly breast and lung cancers. This is especially relevant in Thailand, where these cancers represent leading causes of cancer-related mortality and contribute substantially to national healthcare expenditure [1]. Stratified analyses by service category showed significantly lower daily costs for laboratory, radiological, medical equipment, pharmaceutical, nursing, blood transfusion, and ICU services in the palliative care group compared with the usual care group. These findings are consistent with previous studies from Canada and Thailand demonstrating cost reductions across multiple service categories following palliative care integration [16,19,21,23].

In contrast, room and food costs were higher among patients receiving palliative care, differing from findings reported in a Thai university hospital study, where palliative care reduced these expenditures [21]. This discrepancy may reflect differences in clinical context, as palliative care patients in the present study were more frequently admitted to private rooms to ensure comfort and privacy during end-of-life care. These accommodations incurred higher charges than general ward beds typically used by patients receiving usual care. Cost distribution patterns confirmed this dignity-driven, family-centred approach, characterised by higher proportional spending on room, food, and nursing services alongside reductions in most other categories.

Finally, pre- and post-consultation analyses within the palliative care group demonstrated reductions in total daily hospitalisation costs, laboratory costs, and radiological costs following palliative care consultation. These findings are consistent with previous research from regional hospitals in Thailand, showing reduced healthcare expenditures after palliative care consultation within the final months of life across both inpatient and outpatient settings [22]. These results suggest that palliative care involvement contributes to more efficient resource utilisation. However, increased daily costs for nursing and medical equipment following consultation likely reflect greater resource investment in comfort-focused, person-centred care during the terminal phase.

In interpreting these findings, it is important to note that the comparison between the palliative care and usual care groups relied on unadjusted baseline analyses, which introduces the potential for residual confounding and selection bias. While demographic characteristics were comparable, key clinical factors such as cancer stage, comorbidities, performance status, and do-not-resuscitate status were not adjusted for the models. Consequently, the statistical tests applied in this study were strictly descriptive of observed differences and associations; they do not imply a causal relationship between the type of care received and the resulting costs or resource utilisation. Additionally, given the variations in consultation timing and the small sample sizes in certain cancer-specific subgroups, both the pre- and post-consultation trends and subgroup findings should be interpreted with caution. These preliminary observations highlight the need for future research utilising advanced statistical methods, such as multivariable regression or propensity score matching, to more isolate these effects.

This study has several limitations. First, it was conducted in a single hospital in Thailand, which may limit the generalisability of the findings to other settings. Second, the retrospective nature of the study, reliant solely on hospital records, introduced data incompleteness and potential classification errors; hospital bill records were used as a proxy for costs, which may not fully reflect actual economic costs, and crucial missing data on family meetings, goals-of-care discussions, and advance care planning or do-not-resuscitate decisions limited further evaluation. Third, residual and unmeasured confounding remains a challenge, as the groups were compared primarily on demographics without adjustments for key clinical confounders, including disease severity, performance status, cancer stage, comorbidities, and prognosis, which necessitates a cautious, softened interpretation of causality. Fourth, there is a risk of exposure misclassification and guarantee-time bias, as palliative care operated as a secondary team where the timing and intensity of consultations varied unpredictably based on the readiness of patients, families, and attending physicians, or some patients in the usual care group may have received informal basic palliative approaches without a formal consultation. Fifth, cost-effectiveness in terms of quality of life per unit cost could not be assessed due to the lack of quality-of-life measurements in the medical records, alongside a potential selection bias resulting from the exclusion of pre-terminal hospitalisation consultations.

## 5. Conclusions

This retrospective study demonstrates reductions in daily hospitalisation costs associated with end-of-life cancer care. Palliative care consultation was associated with reductions in more than 50% in direct daily hospitalisation costs for certain cancers, such as breast and lung cancers. Most daily cost components, including laboratory, radiological, medical equipment, pharmaceutical, nursing, blood transfusion, and ICU services, were significantly lower among patients who received palliative care consultation. Future research should examine cost-effectiveness by exploring the relationship between quality of life and healthcare expenditure.

## Figures and Tables

**Table 1:** Demographic characteristics and diagnoses of patients.

Characteristic	Total (N = 266)	Palliative Care Group (N = 154)	Usual Care Group (N = 112)	*p*-Value
Sex [n, (%)]				0.230 *
Female	88 (33.1)	56 (36.4)	32 (28.6)	
Male	178 (66.9)	98 (63.6)	80 (71.4)	
Age (years) [mean ± SD]	65.1 ± 12.8	64.7 ± 12.7	65.6 ± 13.0	0.571 **
Religion [n, (%)]				0.225 *
Buddhism	230 (86.5)	137 (89.0)	93 (83.0)	
Islam	36 (13.5)	17 (11.0)	19 (17.0)	
Health insurance [n, (%)]				0.110 *
UCS	174 (65.4)	101 (65.6)	73 (65.2)	
SSS	12 (4.5)	9 (5.8)	3 (2.7)	
CSMBS	56 (21.1)	35 (22.7)	21 (18.8)	
Other	24 (9)	9 (5.8)	15 (13.4)	
Type of cancer [n, (%)]				0.148 ^†^
Breast	19 (7.1)	12 (7.8)	7 (6.2)	
Colorectal	28 (10.5)	18 (11.7)	10 (8.9)	
Gynaecological	10 (3.8)	9 (5.8)	1 (0.9)	
Haematologic	21 (7.9)	7 (4.5)	14 (12.5)	
Head and neck	14 (5.3)	8 (5.2)	6 (5.4)	
Hepato-pancreatico-biliary	56 (21.1)	31 (20.1)	25 (22.3)	
Lung	82 (30.8)	48 (31.2)	34 (30.4)	
Urological	14 (5.3)	10 (6.5)	4 (3.6)	
Other	22 (8.3)	11 (7.1)	11 (9.8)	

CSMBS: Civil Servant Medical Benefit Scheme, SD: standard deviation, SSS: Social Security Scheme, UCS: Universal Coverage Scheme; * Chi-square test, ** Independent *t*-test, ^†^ Fisher’s exact test.

**Table 2:** Admission and medical utilisation.

	Total (N = 266)	Palliative Care Group (N = 154)	Usual Care Group (N = 112)	*p*-Value
Length of admission (days) [median (IQR)]	6.0 (2.0, 12.0)	8.0 (4.0, 14.8)	3.0 (1.0, 7.0)	**<0.001** *
Intensive care unit				
Yes [n, (%)]	63 (23.7)	13 (8.4)	50 (44.6)	**<0.001** **
Length of utilisation (days) [median (IQR)]	4.0 (2.0, 7.5)	2.0 (2.0, 7.0)	4.0 (2.0, 7.8)	0.890 *
Mechanical ventilator				
Yes [n, (%)]	48 (18.0)	7 (4.5)	41 (36.6)	**<0.001** **
Length of utilisation (days) [median (IQR)]	4.0 (1.8, 8.0)	8.0 (4.5, 10.5)	3.0 (1.0, 8.0)	0.124 *
Cardiopulmonary resuscitation				
Yes [n, (%)]	13 (4.9)	3 (1.9)	10 (8.9)	**0.020** **
Vasopressors and inotropes				
Yes [n, (%)]	40 (15.0)	11 (7.1)	29 (25.9)	**<0.001** **
Surgery				
Yes [n, (%)]	17 (6.4)	8 (5.2)	9 (8.0)	0.496 **
Hospitalisation cost				
Total cost (THB) [median (IQR)]	26,396.2 (11,499.5, 53,332.8)	27,910.8 (13,507.3, 53,208.2)	24,216.4 (9575.4, 53,679.1)	0.305 *
Daily cost (THB) [median (IQR)]	4318.8 (3275.8, 7000.9)	3587.4 (2905.2, 4603.2)	6918.2 (4354.7, 10,773.8)	**<0.001** *

IQR: interquartile range, THB: Thai Baht; * Mann–Whitney U test, ** Chi-square test; Bold *p*-values present statistical significance.

**Table 3:** Daily costs of hospitalisation by type of cancer (in Thai Baht [median (interquartile range)]).

Type of Cancer	Total (N = 266)	Palliative Care Group (N_p_ = 154)	Usual Care Group (N_u_ = 112)	Median Difference (95% CI)	*p*-Value *
Breast (n_p_ = 12, n_u_ = 7)	4013.0 (2762.9, 5473.5)	3326.8 (2602.2, 4119.8)	8049.0 (4949.4, 8654.0)	−4390.1 (−6581.9 to −753.5)	**0.016**
Colorectal (n_p_ = 18, n_u_ = 10)	3514.5 (2711.2, 4861.4)	3422.8 (2501.4, 3722.6)	5595.0 (3701.3, 12,764.0)	−2500.7 (−9862.8 to −711.0)	**0.008**
Gynaecological (n_p_ = 9, n_u_ = 1)	3276.6 (2210.2, 3709.0)	3110.0 (2083.0, 3653.0)	9747.0 (9747.0, 9747.0)	N/A	N/A
Haematologic (n_p_ = 7, n_u_ = 14)	5846.0 (4334.3, 8096.8)	4605.2 (3306.6, 6008.6)	6489.4 (5069.1, 9704.7)	−2097.1 (−6823.9 to 4.2)	0.068
Head and neck (n_p_ = 8, n_u_ = 6)	5069.3 (3566.6, 6424.1)	4129.7 (3381.7, 5604.7)	6272.0 (4463.0, 7455.0)	−1392.5 (−4755.8 to 525.8)	0.107
HPB (n_p_ = 31, n_u_ = 25)	4284.3 (3323.6, 5677.6)	3611.7 (2931.4, 4694.9)	5097.0 (3981.0, 11,310.2)	−1740.0 (−5130.5 to −518.0)	**0.004**
Lung (n_p_ = 48, n_u_ = 34)	4335.4 (3304.9, 7795.8)	3435.4 (3095.6, 4405.5)	7998.7 (4755.4, 12,582.1)	−3896.5 (−5989.4 to −1889.9)	**<0.001**
Urological (n_p_ = 10, n_u_ = 4)	4512.6 (3362.2, 5345.8)	4512.6 (3675.0, 5130.8)	4481.2 (3035.8, 6506.5)	−372.9 (−4206.4 to 2621.1)	0.994
Other (n_p_ = 11, n_u_ = 11)	5419 (3841.9, 8074.8)	3904 (3249.6, 4585.3)	7951.0 (6156.1, 8259.0)	−3281.5 (−4637.8 to 21.1)	0.057

N/A: not available, HPB: Hepato-pancreatico-biliary, 95% CI: 95% confidence interval; n_p_ presents the number of patients in the palliative care group by cancer type. n_u_ presents the number of patients in the usual care group by cancer type. * Mann–Whitney U test; Bold *p*-values present statistical significance.

**Table 4:** Daily costs of hospitalisation by service utilisation categories (in Thai Baht [median (interquartile range)]).

Service Utilisation	Total (N = 266)	Palliative Care Group (N_p_ = 154)	Usual Care Group (N_u_ = 112)	Median Difference (95% CI)	*p*-Value *
Laboratory (n_p_ = 148, n_u_ = 110)	368.5 (182.7, 786.9)	258.8 (113.3, 451.0)	717.8 (371.2, 1204.5)	−417.4 (−546.9 to −302.1)	**<0.001**
Radiology (n_p_ = 143, n_u_ = 103)	220.0 (116.7, 600.0)	157.1 (75.9, 350.0)	370.0 (200.0, 600.0)	−186.7 (−264.3 to −125.3)	**<0.001**
Equipment (n_p_ = 152, n_u_ = 112)	726.8 (388.7, 1373.7)	539.0 (329.4, 760.3)	1231.4 (697.1, 2348.5)	−663.5 (−881.0 to −465.7)	**<0.001**
Pharmaceutical (n_p_ = 154, n_u_ = 112)	410.6 (214.5, 753.5)	305.8 (184.0, 525.9)	686.4 (338.7, 1635.8)	−327.4 (−475.0 to −208.5)	**<0.001**
Nursing service (n_p_ = 154, n_u_ = 112)	736.9 (470.0, 1243.8)	658.6 (436.3, 928.8)	986.7 (596, 2347.9)	−346.7 (−519.8 to −200.6)	**<0.001**
Room and food (n_p_ = 154, n_u_ = 94)	466.8 (400.0, 1500.0)	1066.4 (402.6, 1506.2)	400.0 (287.5, 500.0)	371.9 (220.0 to 600.0)	**<0.001**
Doctor fee (n_p_ = 153, n_u_ = 111)	38.3 (8.3, 233.4)	25.0 (6.2, 233.8)	50.0 (10.8, 215.1)	−1.6 (−7.8 to 3.6)	0.479
Blood transfusion (n_p_ = 44, n_u_ = 47)	396.7 (174.8, 825.0)	275.6 (107.0, 457.0)	595.0 (366.2, 1238.8)	−310.6 (−531.1 to −152.5)	**<0.001**
Intensive care unit (n_p_ = 12, n_u_ = 48)	866.1 (403.0, 1000.0)	343.8 (156.2, 486.1)	1000.0 (666.7, 1000.0)	−518.5 (−750.0 to −295.8)	**<0.001**
Surgery and anaesthesia (n_p_ = 8, n_u_ = 11)	833.3 (80.0, 1114.9)	507.7 (74.4, 879.7)	1050 (219.1, 2230.1)	−446.0 (−2089.7 to 276.1)	0.16
Rehabilitation (n_p_ = 6, n_u_ = 2)	25.9 (17.1, 39.6)	20.5 (13.1, 28.0)	129.2 (88.8, 169.6)	−107.4 (−203.2 to −11.7)	0.07

95% CI: 95% confidence interval; n_p_ presents the number of patients in the palliative care group who utilise the service. n_u_ presents the number of patients in the usual care group who utilise the service. * Mann–Whitney U test; Bold *p*-values present statistical significance.

**Table 5:** Comparison of daily costs of hospitalisation within palliative care group (N = 154) between before and after palliative care consultation (in Thai Baht [median (interquartile range)]).

Service Utilisation	Total	After Palliative Care Consultation Cost	Before Palliative Care Consultation Cost	Median Difference (95% CI)	*p*-Value *
Total daily cost (n_a_ = 154, n_b_ = 100)	3504.0 (2826.3, 4816.6)	3353.6 (2687.9, 4220.4)	3978.1 (3035.8, 5464.5)	−528.7 (−926.5 to −134.8)	**0.008**
Laboratory (n_a_ = 122, n_b_ = 94)	284.2 (129.5, 702.5)	156.2 (69.2, 368.5)	474.5 (270.0, 931.9)	−281.6 (−390.0 to −200.6)	**<0.001**
Radiology (n_a_ = 92, n_b_ = 87)	220.0 (104.5, 600.0)	150.0 (63.0, 300.0)	360.0 (220.0, 800.6)	−211.6 (−315.0 to −147.1)	**<0.001**
Equipment (n_a_ = 151, n_b_ = 88)	550.0 (267.5, 773.8)	607.8 (367.9, 850.0)	363.3 (179.2, 712.5)	202.5 (106.0 to 307.5)	**<0.001**
Pharmaceutical (n_a_ = 154, n_b_ = 99)	284.0 (163.0, 523.8)	305.9 (179.7, 499.9)	220.1 (119.6, 565.2)	39.7 (−16.8 to 93.2)	0.16
Nursing service (n_a_ = 154, n_b_ = 100)	569.2 (416.0, 865.5)	687.1 (430.3, 1035.0)	482.5 (400.0, 657.1)	135.8 (54.2 to 232.0)	**<0.001**
Room and food (n_a_ = 152, n_b_ = 100)	1112.5 (400.0, 1600.0)	1225.0 (400.0, 1600.0)	1000.0 (400.0, 1525.0)	8.5 × 10^−5^ (−25.0 to 37.5)	0.895
Doctor fee (n_a_ = 96, n_b_ = 99)	213.6 (25.0, 237.6)	230.0 (47.5, 230.0)	50 (25.0, 250.8)	−5.6 × 10^−5^ (−12.5 to 30.0)	0.91

95% CI: 95% confidence interval; n_a_ presents the number of patients who utilise the service after palliative care consultation. n_b_ presents the number of patients who utilise the service before palliative care consultation. n_a_ ≠ n_b_ because some patients received palliative care consultation on day one of service, resulting in no prior utilisation data (n_b_). * Mann–Whitney U test; Bold *p*-values present statistical significance.

## Data Availability

The datasets used and/or analysed during the current study are available from the corresponding author on reasonable request due to data privacy restrictions.

## Abbreviations

CPR	cardiopulmonary resuscitation
ICD-10	the 10th revision of the International Classification of Diseases
ICU	intensive care unit
IQR	interquartile range
LMICs	low- and middle-income countries
SD	standard deviation
THB	Thai Baht

## References

[1] Ministry of Public Health (2024). Public Health Statistics A.D. https://spd.moph.go.th/wp-content/uploads/2025/11/สถิติสาธารณสุข-2567_final.pdf.

[2] World Health Organization (2024). Global Cancer Burden Growing, Amidst Mounting Need for Services. https://www.who.int/news/item/01-02-2024-global-cancer-burden-growing--amidst-mounting-need-for-services.

[3] Alawneh A., Anshasi H. (2021). Place of death for patients treated at a tertiary cancer center in Jordan. Support Care Cancer.

[4] Alshemmari S.H., Elbasmi A.A., Alsirafy S.A. (2015). The place of death of patients with cancer in Kuwait. BMJ Support Palliat. Care.

[5] Bekelman J.E., Halpern S.D., Blankart C.R., Bynum J.P., Cohen J., Fowler R., Kaasa S., Kwietniewski L., Melberg H.O., Onwuteaka-Philipsen B. (2016). Comparison of site of death, health care utilization, and hospital expenditures for patients dying with cancer in 7 developed countries. JAMA.

[6] Ferreira P.C., Gomes B.J.O., Canato G.M., Mantovani E.B., de Lima L.V., Pavinati G., Lino I.G.T., Marcon S.S. (2025). Mortality from malignant neoplasms at home and in hospitals in Brazil, 2002–2022: Sociodemographic characteristics and temporal trends. Rev. Bras. Epidemiol..

[7] Shalev Many Y., Shvartzman P., Wolf I., Silverman B.G. (2023). Place of death for Israeli cancer patients over a 20-year period: Reducing hospital deaths, but barriers remain. Oncologist.

[8] Yang C.H., Wu C.Y., Cheng S.Y., Mori M., Suh S.-Y., Kim S.-H., Lin W.-Y., Yamaguchi T., Huang H.-L., Hamano J. (2025). Reasons for and congruence between preferred and actual place of death among cancer patients receiving end-of-life care: A cross-cultural multicenter prospective cohort study in East Asia. Cancers.

[9] Guzzinati S., Andreotti A., Lopez T., Gori S., Gagliani A., Mallone S., Pierannunzio D., Tavilla A., Buja A., Zorzi M. (2025). Cost profiles of cancer patients at the end of life: Estimates from the EPICOST-study. PLoS ONE.

[10] Kang D.W., Shim Y.B., Lee E.K., Park M.H. (2022). Healthcare resource utilization and medical costs in patients with terminal cancer during best supportive care. PLoS ONE.

[11] Chen S., Cao Z., Prettner K., Kuhn M., Yang J., Jiao L., Wang Z., Li W., Geldsetzer P., Bärnighausen T. (2023). Estimates and projections of the global economic cost of 29 cancers in 204 countries and territories from 2020 to 2050. JAMA Oncol..

[12] World Health Organization (2020). Palliative Care. https://www.who.int/news-room/fact-sheets/detail/palliative-care.

[13] Gwyther L., Bates M.J., Tran B., Grant L., Harding R., Krakauer E.L., May P., Namisango E., Rajagopal M.R., Reid E. (2026). Economic benefits of investment in palliative care: An appraisal of current evidence and call to action. J. Pain Symptom Manag..

[14] Hashimoto Y., Hayashi A., Teng L., Igarashi A. (2021). Real-world cost-effectiveness of palliative care for terminal cancer patients in a Japanese general hospital. J. Palliat. Med..

[15] Hua M., Lu Y., Ma X., Morrison R.S., Li G., Wunsch H. (2020). Association between the implementation of hospital-based palliative care and use of intensive care during terminal hospitalizations. JAMA Netw. Open.

[16] Isenberg S.R., Meaney C., May P., Tanuseputro P., Quinn K., Qureshi D., Saunders S., Webber C., Seow H., Downar J. (2021). The association between varying levels of palliative care involvement on costs during terminal hospitalizations in Canada from 2012 to 2015. BMC Health Serv. Res..

[17] Kremenova Z., Svancara J., Kralova P., Moravec M., Hanouskova K., Knizek-Bonatto M. (2022). Does a hospital palliative care team have the potential to reduce the cost of a terminal hospitalization? A retrospective case-control study in a Czech tertiary university hospital. J. Palliat. Med..

[18] Yadav S., Heller I.W., Schaefer N., Salloum R.G., Kittelson S.M., Wilkie D.J., Huo J. (2020). The health care cost of palliative care for cancer patients: A systematic review. Support Care Cancer.

[19] Chansriwong P., Thokanit N.S., Semsarn S., Utthiya P., Kasemsup V. (2023). Cost savings associated with palliative care among patients with genitourinary malignancies. J. Clin. Oncol..

[20] Sinsuwan W.P.S., Gomutbutra P., Kongkum K., Kosuwon W. (2016). A retrospective, single center, observational study, comparing the direct cost of end-of-life care patients with advanced cancer care: Palliative care versus usual care. J. Palliat. Care Med..

[21] Thokanit N.S., Chansriwong P., Semsarn S., Kasemsup V., Piebpien P., Sirachainan E. (2025). Comparison of direct medical costs between integrated care with palliative care and standard treatment in older patients with cancer. Nurs. Res. Inno.

[22] Wajatieng W. (2025). Reducing healthcare costs through early palliative care in terminal cancer: Evidence from a retrospective cohort study. J. Nakornping Hosp..

[23] Chanthong P., Punlee K., Kowkachaporn P., Intharakosum A., Nuanming P. (2023). Comparison of direct medical care costs between patients receiving care in a designated palliative care unit and the usual care units. Asia Pac. J. Clin. Oncol..

[24] Kanjanopas T. (2022). Development of palliative care in Thailand. Thai Health Promot J..

[25] Husereau D., Drummond M., Augustovski F., de Bekker-Grob E., Briggs A.H., Carswell C., Caulley L., Chaiyakunapruk N., Greenberg D., Loder E. (2022). Consolidated Health Economic Evaluation Reporting Standards 2022 (CHEERS 2022) Statement: Updated reporting guidance for health economic evaluations. Value Health.

[26] Vandenbroucke J.P., von Elm E., Altman D.G., Gøtzsche P.C., Mulrow C.D., Pocock S.J., Poole C., Schlesselman J.J., Egger M., STROBE initiative (2007). Strengthening the Reporting of Observational Studies in Epidemiology (STROBE): Explanation and elaboration. Ann. Intern Med..

[27] National Statistical Office, Ministry of Digital Economy and Society (2025). Thai Economic Indicators 2025.

[28] Worldwide Palliative Care Alliance (2014). Global Atlas of Palliative Care at the End of Life.

[29] Tripodoro V.A., Fidalgo J.F.L., Pons J.J., Connor S.R., Garralda E., Bastos F., Montero Á., Llamas L.M., Béjar A.C., Suárez D. (2025). First-ever global ranking of palliative care: 2025 world map under the new WHO framework. J. Pain Symptom Manag..

[30] Ratjaroenkhajorn S. A Review of Hospice Care in Thailand. https://en.peacefuldeath.co/a-review-of-hospice-care-in-thailand/.

[31] Davis M.P., Van Enkevort E.A., Elder A., Young A., Ordonez I.D.C., Wojtowicz M.J., Ellison H., Fernandez C., Mehta Z. (2022). The influence of palliative care in hospital length of stay and the timing of consultation. Am. J. Hosp. Palliat. Care.

[32] Matthews S., Hurley E., Johnston B.M., Kane P., Ryan K., Tiernan E., Normand C., May P. (2024). Does a palliative medicine service reduce hospital length of stay and costs in adults with a life-limiting illness?—A difference-in-differences evaluation of service expansion in Ireland. Ann. Palliat. Med..

[33] Yeh J.C., Urman A.R., Besaw R.J., Dodge L.E., Lee K.A., Buss M.K. (2022). Different associations between inpatient or outpatient palliative care and end-of-life outcomes for hospitalized patients with cancer. JCO Oncol. Pract..

[34] Bajwah S., Oluyase A.O., Yi D., Gao W., Evans C.J., Grande G., Todd C., Costantini M., E Murtagh F., Higginson I.J. (2020). The effectiveness and cost-effectiveness of hospital-based specialist palliative care for adults with advanced illness and their caregivers. Cochrane Database Syst. Rev..

